# Rate-Compatible LDPC Codes for Continuous-Variable Quantum Key Distribution in Wide Range of SNRs Regime

**DOI:** 10.3390/e24101463

**Published:** 2022-10-13

**Authors:** Xiaodong Fan, Quanhao Niu, Tao Zhao, Banghong Guo

**Affiliations:** 1 Laboratory of Nanophotonic Functional Materials and Devices, South China Normal University, Guangzhou 510006, China; 2 Laboratory of Quantum Engineering and Quantum Materials, South China Normal University, Guangzhou 510006, China; 3 National Quantum Communication (Guangdong) Co., Ltd., Guangzhou 510700, China; 4 Key Laboratory of Quantum Information, University of Science and Technology of China, Chinese Academy of Sciences, Hefei 230026, China

**Keywords:** rate compatible, LDPC, continuous-variable quantum key distribution, wide range of SNRs regime

## Abstract

Long block length rate-compatible low-density parity-compatible (LDPC) codes are designed to solve the problems of great variation of quantum channel noise and extremely low signal-to-noise ratio in continuous-variable quantum key distribution (CV-QKD). The existing rate-compatible methods for CV-QKD inevitably cost abundant hardware resources and waste secret key resources. In this paper, we propose a design rule of rate-compatible LDPC codes that can cover all potential SNRs with single check matrix. Based on this long block length LDPC code, we achieve high efficiency continuous-variable quantum key distribution information reconciliation with a reconciliation efficiency of 91.80% and we have higher hardware processing efficiency and lower frame error rate than other schemes. Our proposed LDPC code can obtain a high practical secret key rate and a long transmission distance in an extremely unstable channel.

## 1. Introduction

The cryptosystem based on computational complexity is being challenged by increasingly developed quantum computation. Quantum key distribution (QKD) [1,2,3,4], being one-time pad, has been one of the best solutions for its absolute security. QKD enables two remote separated parties named Alice and Bob to extract a symmetrical string of secret keys using a quantum channel.

Currently, there are mainly two types of QKD protocols, called discrete-variable QKD (DV-QKD) [5] and continuous-variable QKD (CV-QKD) [6,7]. In DV-QKD, the information is coded on discrete variables of finite dimensional Hilbert space, such as the polarization or phase of single photon state. In CV-QKD, the information is coded on continuous variables of an infinite-dimensional Hilbert space, including the regular component of coherent state. Compared with the single photon detector used in DV-QKD, homodyne or heterodyne detection techniques, which are used to measure the transmitted quantum states, have already been applied in classical optical communication. Therefore, CV-QKD has great practical advantages for its low cost because of the relatively mature development and being able to transmit in common fiber with classical optical communication. Furthermore, CV-QKD can achieve higher capacity with frequency-multiplexed entanglement source [8].

Due to the imperfection of the quantum channel and potential eavesdropper Eve, the key strings held by Alice and Bob are not consistent, so that a procedure called post-processing is necessary to make them identical. The post-processing of CV-QKD mainly includes four steps: base vector comparison, parameter estimation, information reconciliation and privacy amplification. Information reconciliation is the most important part, whose performance has a direct correlation to the secret key rate. One of the major factors in information reconciliation is reconciliation efficiency β, which is given by β=R/C. The *R* is the rate of key and C=0.5log(1+SNR) is the channel compacity. The hardware processing efficiency $\alpha =D_{out}/D_{in}$, where $D_{in}$ represents the data that are input to the hardware device (e.g., Field-programmable Gate Array, FPGA and Graphics Processing Unit, GPU) during information reconciliation and $D_{out}$ represents the output data in unit time [9]. $I_{AB}$ is the mutual information between Alice and Bob. $\chi_{BE}$ is the Holevo bound, which is the maximal bound on the information available to the eavesdropper. The factors mentioned above are used to evaluate the performance in a frame, while frame error rate (FER) represents the failure probability of the frames. Ultimately, the practical secret key rate *K* is given by

(1) $$K=\alpha \left(1-FER\right)\left(\beta I_{AB}-\chi_{BE}\right).$$

The parameters mentioned above is related to the error correcting codes, among them low-density parity-compatible (LDPC) code is efficient for CV-QKD [10]. The LDPC code obtained by good degree distribution and reasonable construction method has good error correction performance. The crux of designing a LDPC code is to construct a check matrix which includes check nodes and variable nodes. The degree distribution of check node ρ(x) and variable node λ(x) are expressed as:

(2) $$\rho \left(x\right)=\sum_{j=2}^{d_{c}}\rho_{i}x^{j-1}$$

(3) $$\lambda \left(x\right)=\sum_{i=2}^{d_{v}}\lambda_{i}x^{i-1},$$

$\rho_{i}/\lambda_{i}$ is the proportion of the number of edges owned by the check/variable node with degree j/i to the total number of edges in the Tanner graph and $d_{c}/d_{v}$ indicates the maximum degree of the check/variable node.

However, quantum is easily influenced in the process of quantum signal preparation and transmission. To realize the free space QKD with satellite [11,12], ship [13], unmanned aerial vehicles [14] or those with orbital angular momentum, we have to take mode distortion, beam wander, weather etc. into account. Therefore, the problems of great variation of quantum channel noise and extremely low signal-to-noise ratio (SNR) have to be solved.

One of the simplest rate-compatible methods for LDPC code is to operate on single-matrix using puncturing, shortening and extending. Furthermore, Gao proposed multi-matrix rate-compatible reconciliation where, in each iteration, multiple matrices produce more useful information to correct errors such that the iteration number falls and the convergence speed increases [15]. However, it inevitably decreases the performance of the original check matrix. Another commonly used way is to construct several check matrices with different code rates to meet the requirements of different SNRs. However, for CV-QKD, the code length has to be longer than 100,000. Base matrices are at least 64,800 long even when we construct the spatially coupled (SC)-LDPC codes or quasi-cyclic (QC)-LDPC codes [16]. As one of the most effective decoding tools of LDPC code, the FPGA has limited hardware resources. To realize high efficiency information reconciliation with FPGA in an extremely unstable channel, it is necessary to construct a single-matrix rate-compatible error correction code. A comparison of the existing works with our proposed LDPC code is shown in Table 1.

In this paper, we first obtain degree distribution with discrete density evolution and differential evolution algorithm. Then we use random construction, progressive edge growth (PEG) algorithm and rate compatible methods of extending and puncturing to construct a check matrix with a code length of 64,800. Finally, we extend the above LDPC code with quasi-cyclic extension to a code length of 648,000. The results show that the proposed codes have a reconciliation efficiency of 91.80%, higher hardware processing efficiency and lower FER than other schemes. Therefore, we can obtain a high practical secret key rate and a longer transmission distance in an extremely unstable channel with wide range of SNRs.

The remainder of this paper is organized as follows. In Section 2, we present some preliminaries of LDPC codes and rate-compatibility. In Section 3, we introduce how to construct our rate compatible (RC)-LDPC code. In Section 4, we present the simulation results and comparisons for the proposed scheme and existing schemes. Finally, the conclusions are drawn in Section 5.

## 2. Preliminaries

In this section, we first briefly introduce the discrete density evolution and differential evolution, which are used to generate degree distribution. Then we introduce the constructions: random construction, PEG algorithm and QC-LDPC extension, with which we can construct the check matrix with the degree distribution ahead. We also introduce the rate compatible methods: puncturing and extending.

### 2.1. Methods of Obtaining Degree Distribution

#### 2.1.1. Discrete Density Evolution

Compared with continuous density evolution [17] and Gaussian approximation algorithm [18], discrete density evolution [19] has lower complexity and higher accuracy. Therefore, in this paper, we use discrete density evolution to obtain the optimal degree distribution of LDPC codes. The main steps are as follows:

- We firstly define two functions: quantized function *Q* and probability mass function *S*.
  (4) $$Q\left(x\right)=\left\{\begin{array}{cc} \left\lfloor \frac{x}{\Delta }+\frac{1}{2}\right\rfloor , & x\geq \frac{\Delta }{2}\\ \left\lceil \frac{x}{\Delta }-\frac{1}{2}\right\rceil , & x\leq -\frac{\Delta }{2},\\ 0, & else \end{array}\right.$$
  $\left\lfloor x\right\rfloor$ is the largest integer not greater than *x*; and $\left\lceil x\right\rceil$ is the smallest integer not less than *x*. The value range of decoded message is [−L,L] and evenly divided into $m=2^{q}$ intervals; the quantization interval Δ is given by 2L/m.
  (5) $$S\left(P_{a},P_{b}\right)=\sum_{\left(i,j):k\Delta =R(i\Delta ,j\Delta \right)}P_{a}\left[i\right]\cdot P_{b}\left[j\right].$$
  In which two-input operator *R* is
  (6) $$R\left(a,b\right)=Q(\tanh^{-1}\left(tanh\frac{a}{2}tanh\frac{b}{2}\right)),$$
  where *a* and *b* are quantized messages.
- The check node and variable node updating of discrete density evolution is
  (7) $$p_{\overset{-}{u}}^{\left(l\right)}=\sum_{i=2}^{d_{r}}\rho_{i}S^{i-1}\left(p_{\overset{-}{v}}^{\left(l-1\right)}\right)$$
  (8) $$p_{\overset{-}{v}}^{\left(l\right)}\left(k\right)=p_{\overset{-}{v}}^{\left(0\right)}\left(k\right)\cdot \sum_{i=2}^{d_{v}}\lambda_{i}{⊗}^{i-1}{\left(p\right.}_{\overset{-}{u}}^{\left(l\right)}\left(k\right)),$$
  ⨂ is discrete convolution and *l* is the iteration number. The initial value $p_{\overset{-}{v}}^{\left(0\right)}$ is
  (9) $$\left.p_{\overset{-}{v}}^{\left(0\right)}=\frac{\sigma }{8\pi }exp\left(-\frac{{\left(2-\sigma^{2}v\right)}^{2}}{8\sigma^{2}}\right),v^{\left(0\right)}\right.∼N\left(\frac{2}{\sigma^{2}},\frac{4}{\sigma^{2}}\right).$$
- Finally, we calculate the error rate with
  (10) $$p_{\overset{-}{e}}^{\left(l\right)}=p_{\overset{-}{v}}^{\left(l\right)}\left(0\right)+\sum_{k=-m/2}^{-1}p_{\overset{-}{v}}^{\left(l\right)}\left(k\right).$$
  End the procedure when the $p_{\overset{-}{e}}^{\left(l\right)})<0$ or *l* reaches the maximum number of iterations. Otherwise, we continue to update the check node and variable node.

Discrete density evolution is first proposed to obtain the noise threshold according to the degree distribution $\rho_{i}$ and $\lambda_{i}$. In our work, we use it to obtain the degree distribution under specific channel noise.

#### 2.1.2. Differential Evolution

Stom first proposed the differential evolution algorithm in 1995 to solve the optimization problem [20]. It uses differential mutation operator and crossover operator to generate new individuals by the way of survival of the fittest. Based on this method, we can obtain the optimal degree distribution under specific channel noise.

- Set channel noise threshold σ, target error probability $P_{e}$, maximum number of iterations $l_{max}$, maximum degree of variable node $d_{v}$ and the number of terms of degree distribution polynomial *n*.
- Randomly generate NP vectors $P_{i,G},i=1,2,\ldots ,NP$ for the degree distribution of variable node. Use discretized density to evolve each vector and obtain the respective error probability $P_{e_{i},G}$. The vector with the lowest error probability is marked as the best vector $P_{best,G}$ and its error probability is marked as $P_{e_{best},G}$.
- For each *i*, randomly choose four vectors from set of $P_{i,G}$ and the new vector is updated by
  (11) $$v_{i,G+1}=P_{best,G}+0.5\left(P_{r_{1},G}-P_{r_{2},G}\right)+0.5\left(P_{r_{3},G}-P_{r_{4},G}\right).$$
  Calculate the corresponding error probability $P_{v_{i},G+1}$ for each new vector $v_{i,G+1}$.
- For each *i*, compare $P_{e_{i},G}$ with $P_{v_{i},G+1}$ and let $P_{i,G+1}=v_{i,G+1}$ if $P_{e_{i},G}>P_{v_{i},G+1}$. The vector with the lowest error probability is marked as the best vector $P_{best,G+1}$ and its error probability is marked as $P_{e_{best},G+1}$.
- If the error probability corresponding to the best vector $P_{e_{best},G+1}>P_{e}$, update the vectors again and return to step (4). If $P_{e_{best},G+1}\leq P_{e}$, the $P_{best,G+1}$ is the ideal vector that we want.

### 2.2. Constructions

In this work, we use random construction, the PEG algorithm and QC extension for their good results in various situations.

#### 2.2.1. Random Construction

Various random constructions have been proposed based on the same core thought, that is, place non-zero elements in random unfilled positions in the check matrix without violating any set constraint. There are two constraint rules: one is that line $l_{i}$ contains $X_{i}$ “1” and column $c_{i}$ contains $Y_{i}$ “1” according to the degree distribution of check nodes and variable nodes; the other one is the number of elements “1” at the same position in any two rows or columns is less than or equal to 1. It means that the shortest girth has to be longer than 4.

#### 2.2.2. Progressive-Edge-Growth Algorithm

**Figure 1:**
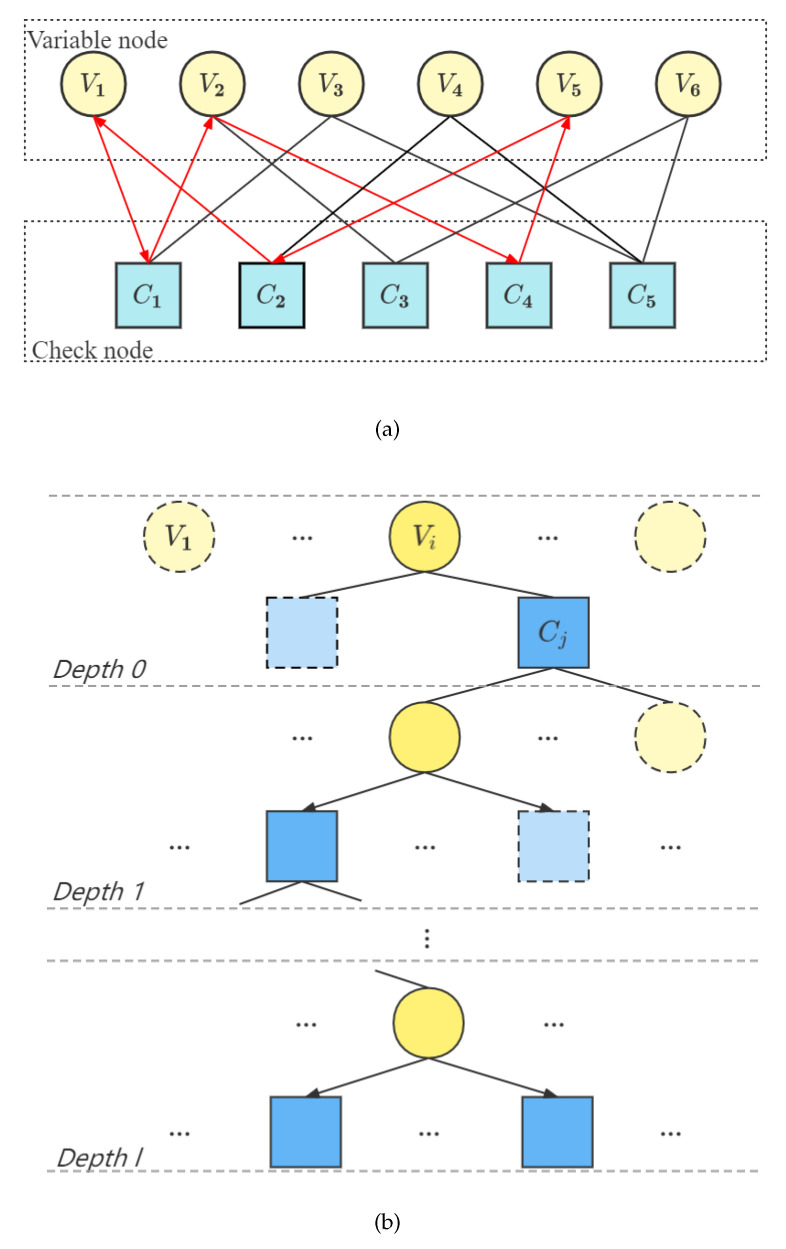
(**a**) Tanner graph; (**b**) PEG algorithm.

Before introducing the PEG algorithm, we first introduce a common representation of LDPC codes—the Tanner diagram and several concepts. As shown in Figure 1a, $V_{i}$ is a variable node, $C_{j}$ is a check node and the line between them is called an edge. If two nodes are connected with each other, we say these two nodes are adjacent to each other. The girth is defined as the minimum number of lines that comes from a node and back to this node, whose intermediate node is only passed once. As shown in Figure 1a, the shortest girth is 6 and one of them is $V_{1}\to C_{1}\to V_{2}\to C_{4}\to V_{5}\to C_{2}\to V_{1}$, for instance.

For the PEG algorithm, new edges are added to make the loop girth in the Tanner diagram corresponding to the check matrix as large as possible. As shown in Figure 1b, the steps are as follows:

- Determine the number of check node, variable node and the degree distribution of variable node.
- Randomly choose a variable node $V_{i}$ and find the check node $C_{j}$ with the least number of connected edges in the Tanner graph. Then connect the variable node $V_{i}$ and the check node $C_{j}$ with an edge and take it as the first edge of the variable node $V_{i}$.
- Take the variable node $V_{i}$ as the root node and expand the current Tanner diagram. When the expansion depth is *l*, the set of check nodes adjacent to $V_{i}$ is recorded as $N_{V_{i}}^{l}$. The $\bar{N_{V_{i}}^{l}}$ is the complement set of $N_{V_{i}}^{l}$, where the complete set $V_{c}$ is the set of all variable nodes. Expand the Tanner graph with the root node and the depth of *l*. When $\bar{N_{V_{i}}^{l}}\neq ⌀$, $\bar{N_{V_{i}}^{l+1}}=⌀$ and the number of nodes contained in $N_{V_{i}}^{l}$ stops increasing but is still less than the number of matrix rows *l*, connect the check node $C_{j}$ with the least number of connected edges to the variable node $V_{i}$.
- Repeat step (2) to add edges to the selected variable nodes until all of them are added.
- Repeat steps (1) to (3) to add edge for all other variable nodes.

#### 2.2.3. QC-LDPC Extension

QC-LDPC extension is uniquely determined by the dimension and shift times of the circulant matrix. Its quasi-cyclic characteristics make the process of coding and decoding more efficient. Compared with randomly constructed LDPC codes, QC-LDPC codes have lower error level and are more convenient for storage and hardware implementation. We multiply the corresponding positions of the base matrix $H_{b}$ and the coefficient matrix $H_{c}$ and we define this operation as ⊙, the expression is expressed as follows:

(12) $$H_{b}⊙H_{c}=\left[\begin{array}{ccc} B_{1,1} & \cdots & B_{1,i}\\ \vdots & \ddots & \vdots \\ B_{j,1} & \cdots & B_{j,i} \end{array}\right]⊙\left[\begin{array}{ccc} C_{1,1} & \cdots & C_{1,i}\\ \vdots & \ddots & \vdots \\ C_{j,1} & \cdots & C_{j,i} \end{array}\right]=\left[\begin{array}{ccc} B_{1,1}C_{1,1} & \cdots & B_{1,i}C_{1,i}\\ \vdots & \ddots & \vdots \\ B_{j,1}C_{j,1} & \cdots & B_{j,i}C_{j,i} \end{array}\right].$$

Take lifting size of 3 as an example, the elements of the base matrix are 0 and 1, and the elements of the coefficient matrix are 1, 2 and 3. Then the matrix elements are replaced by the cyclic permutation matrices (CPMs). We replace 0 with zero matrices, 1 with $\left[\begin{array}{ccc} 1 & 0 & 0\\ 0 & 1 & 0\\ 0 & 0 & 1 \end{array}\right]$, 2 with $\left[\begin{array}{ccc} 0 & 1 & 0\\ 0 & 0 & 1\\ 1 & 0 & 0 \end{array}\right]$ and 3 with $\left[\begin{array}{ccc} 0 & 0 & 1\\ 1 & 0 & 0\\ 0 & 1 & 0 \end{array}\right]$.

### 2.3. Methods of Rate-Compatible

**Figure 2:**
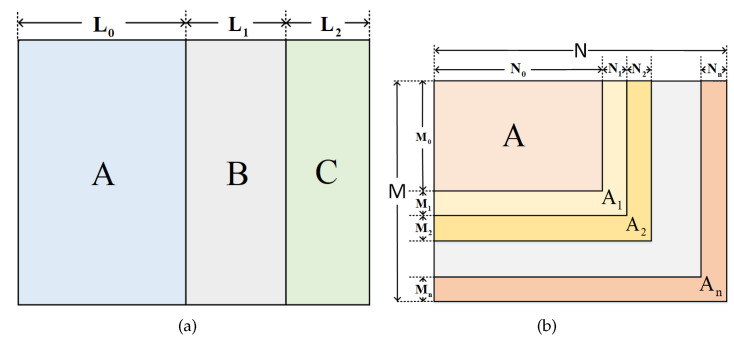
The rate-compatible method: (**a**) puncturing; (**b**) extending.

Puncturing is a method that makes the code rate change from low to high. As shown in Figure 2a, the submatrix A are information bits and submatrix B and C are check bits. The initial code rate is $R=L_{0}/\left(L_{0}+L_{1}+L_{2}\right)$. By deleting the submatrix C, we can obtain a code rate increasing to $R=L_{0}/\left(L_{0}+L_{1}\right)$.

On the contrary, extending as shown in Figure 2b enables the code rate to change from high to low. We first construct a check matrix A with the high bit rate of $\left(N_{0}-M_{0}\right)/N_{0}$. Moreover, by adding the submatrix $A_{n}$, we extend the matrix to make it compatible for the low rate. The code rate is expressed as:

(13) $$R_{i}=\frac{\sum_{0}^{n}N_{i}-\sum_{0}^{n}M_{i}}{N}.$$

## 3. Proposed Check Matrix for RC-LDPC Codes with Wide Range of SNRs Regime

From the Equation (1) we can see that high hardware processing efficiency and reconciliation efficiency result in a good performance of final secret key rate for a given SNR. Proper degree distribution and reasonable construction method lead to good error correction performance.

### 3.1. Obtaining Degree Distribution

We first obtain the initial optimal degree distribution using discretize density evolution and differential evolution refer to Section 2.1.1 and Section 2.1.2. Maximum degree of variable node and the number of terms of degree distribution polynomial are set as 10 and 4, respectively.

From the initial optimal degree distribution, we find that the pairs of degree distribution are distributed nearby $\lambda_{3}$ and $\lambda_{7}$ except of $\lambda_{2}$ and $\lambda_{10}$. Therefore, we calculate the average number of $\lambda_{3}$ and $\lambda_{7}$ at rate from 0.3 to 1, i.e., SNR from 0.1 to 3 (the degree distribution is appropriate to the SNR larger than 3 but the maximum rate 1 corresponds to the SNR of 3). The initial values are average number $\bar{\lambda_{3}}$ and $\bar{\lambda_{7}}$, and maximum degree of variable node and the number of terms of degree distribution polynomial are still set as 10 and 4. The difference is that the degree distribution of the variable distribution is set on the $\lambda_{2}$, $\lambda_{3}$, $\lambda_{7}$ and $\lambda_{10}$ instead of a random distribution. Then we repeat the above operations to obtain the optimal degree distribution in these conditions.

Through the above operations, we obtain the degree, the maximum degree of the variable node, and the number of terms of the degree distribution polynomial. Ultimately, we calculate the optimal degree distribution for proposing our LDPC code with Algorithm 1.
**Algorithm 1:** Obtaining the ultimate variable degree distribution with density evolution and differential evolution **Input:** Target error probability $P_{e}$, maximum number of iterations $l_{max}$, population size NP=50, the number of terms of variable node degree distribution polynomial l=5, the highest power of variable node degree distribution, $\lambda_{3}=0.0047$, $\lambda_{7}=0.5072$ **Output:** Error rate $P_{e_{best}}$, vector $P_{best}$1:**for***i* = 1 to NP **do**2:    refer to Section 2.1.1 generate vector $P_{i}$ with $\lambda_{2}$, $\lambda_{3}$, $\lambda_{7}$, $\lambda_{8}$ and $\lambda_{10}$, $\lambda_{2}+\lambda_{8}+\lambda_{10}=0.4881$;3:    calculate the error probability $P_{e_{i}}$;4:    **if** $P_{e_{best}}>P_{e_{i}}$ **then**5:        $P_{e_{best}}\leftarrow P_{e_{i}};P_{best}\leftarrow P_{i}$;6:    **end if**7:**end for**8:**for**j=1 to $l_{max}$ **do**9:    randomly choose four numbers $r_{1}$, $r_{2}$, $r_{3}$, $r_{4}$ from 1 to NP;10:    $v_{j}$ = $P_{best}$ + $0.5(P_{r_{1}}$ - $P_{r_{2}}$ + $P_{r_{3}}$ - $P_{r_{4}})$;11:    calculate the error probability $P_{e_{j}}$;12:    **if** $P_{e_{best}}<P_{e}$ **then**13:        output $v_{j}$;14:    **end if**15:    **if** $P_{e_{best}}>P_{e_{j}}$ **then**16:        $P_{e_{best}}\leftarrow P_{e_{j}}$;17:    **end if**18:**end for**

Table 2 is the result of Algorithm 1, whose input signal X∼(0,1) and additive white Gaussian noise $Z∼(0,\sigma^{2})$ are random variables that obey Gaussian distribution and independent of each other. The channel noise $SNR=1/\sigma^{2}$ and σ represents the maximum allowed value of noise for the additive white Gaussian channel. For ρ(x)=λ(x)=1, the check node degree distribution is definite with the constraint condition $r=1-\int_{0}^{1}\rho (x)dx/\int_{0}^{1}\lambda (x)dx$. The degree distribution in our scheme especially decreases the difficulty of constructing the check matrix.

In order to maximize the use of limited key resources, we still need to fully consider the condition of rate lower than 0.1. Obviously, the secret key rate is low for the low mutual information $I_{AB}$. Therefore, in order to simplify our work, the degree distribution pairs we choose for the rate lower than 0.1 are directly refer to Appendix A [21,22].

### 3.2. Constructing Check Matrix for RC-LDPC Code

**Figure 3:**
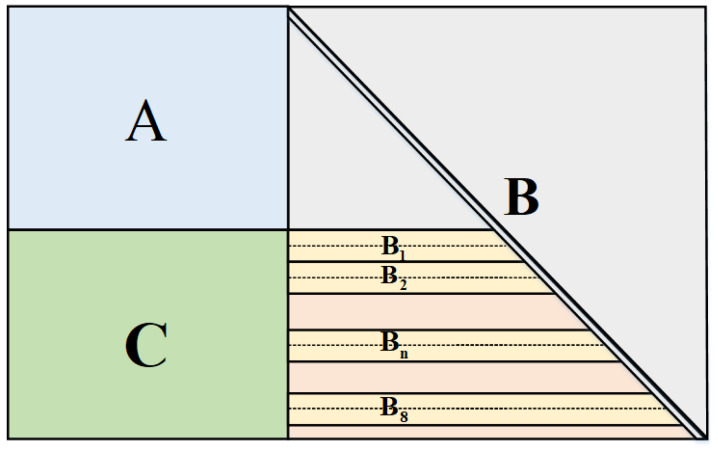
The check matrix for RC-LDPC codes with wide range of SNR.

With the degree distribution we obtained above, we construct a single matrix RC-LDPC code simultaneously with the random construction, the PEG algorithm, and QC-LDPC codes mentioned in Section 2. The structure of the check matrix is shown in Figure 3 and combined with parts A, B and C.

The part A is a shared part for the rate from 0.1 to 1, which is constructed with $\bar{\lambda_{3}}$ and $\bar{\lambda_{7}}$. This structure has the advantage of reducing computational complexity and saving the storage resources. Previous work showed that the PEG algorithm has better performance at SNR∼3 [23], while random construction exhibits better performance at SNR∼1 [24]. Therefore, the construction that we use to construct the sub-matrix A is the PEG algorithm.

The part B is constructed with rest of degree distribution to realize the rate-compatible method of puncturing. In order to further improve the performance of our LDPC code, we construct the check matrix with the thought of puncturing. More specifically, we divide submatrix $B_{n}$ into two part and construct one part when the *R* decreases every 0.05. For rate from 0.3 to 0.1, this number is 0.1. We use PEG algorithm to construct $B_{1}$ to $B_{5}$ and random construction to construct extra part. Moreover, the structure of part B is a lower triangular matrix, which can be directly encoded.

Multi-edge-type (MET)-LDPC codes are employed with low SNRs due to their good error-correcting performances, more amenable decoding complexity and also being able to be rate-compatible at low rates [25]. Based on the check matrix above, we construct part C with degree distribution of the MET-LDPC codes from Appendix A for the rate from 0.01 to 0.1.

## 4. Simulation Experiment

In this section, we summarize the implementation results of the proposed LDPC codes over an unstable channel. Our purpose is to construct a RC-LDPC code with single matrix that can be adapt to the SNR from 0.01 to 15. We show the performance of reconciliation efficiency β, hardware processing efficiency α and FER, which are influenced by the change of SNR. Furthermore, the decoding algorithm is a modified Min-Sum algorithm.

**Figure 4:**
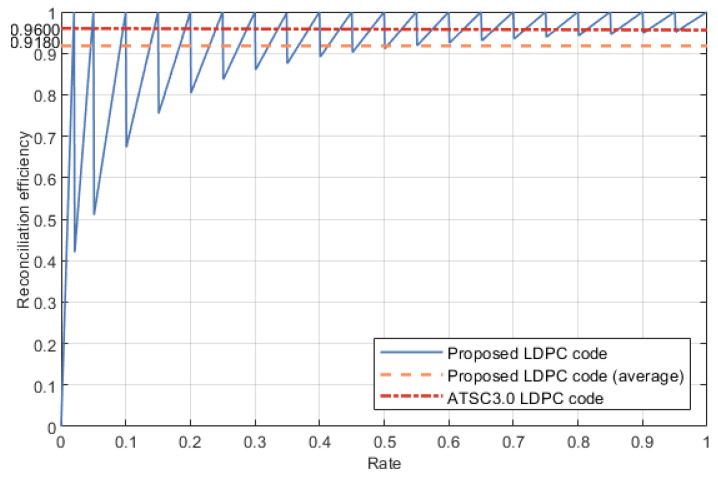
The reconciliation efficiency for different code rate.

The reconciliation efficiency comes from β=R/C. Referring to the construction mentioned in Section 3.2, we change the check matrix when *R* reduces to a certain extent. When *R* is from 0.3 to 1, *C* decreases 0.1 to an integer multiple of 0.1. When *R* is from 0.01 to 0.3, *C* decreases 0.05 to an integer multiple of 0.05. In Figure 4, assuming that the channel noise is uniformly distributed, the LDPC code we proposed has an average reconciliation efficiency β of 91.80%, and for higher rates from 0.3 to 1 this number is 96.13%. Because the data with rate lower than 0.3 only have a little contribution to reconciliation efficiency, the practical reconciliation efficiency is close to 96.13%. Compared with the existing scheme, the proposed LDPC code has a relatively high reconciliation efficiency.

From Equation (1), the secret key rate is also related to the hardware processing efficiency α, which is equal to the ratio of $D_{out}$ and $D_{in}$. More specifically, supposing the times used to load check matrix, load data and decode data are $t_{lm}$, $t_{ld}$ and $t_{dd}$, separately. The number of times that check matrix has to be reloaded is *n* and the number of data blocks that have to be processed is *m*. Suppose the secret key rate that optical system can provide is *M*, the number of data blocks *m* is M/L. The hardware processing efficiency α is

(14) $$\alpha =\frac{1}{nt_{lm}+m\left(t_{ld}+t_{dd}\right)}$$

**Figure 5:**
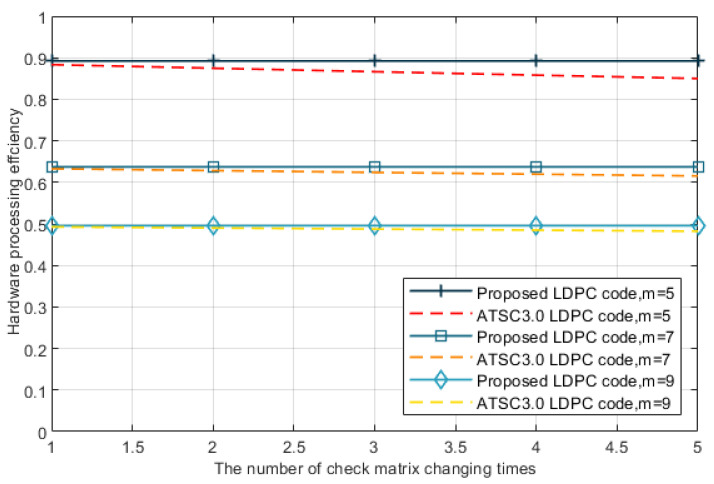
The hardware processing efficiency α influenced by the number of check matrix changing times *n*.

Because of the finite-size effects, the block length in the procedure of privacy amplification is at least $10^{7}$, which also takes up abundant hardware resource [26,27], so that not all the check matrices can be stored in advance. The reconciliation efficiency will be reduced quickly even if the SNR changes in a very small range. Therefore, other schemes have to reload the appropriate check matrix and then load and decode data when the rate is higher than the channel capacity. With our proposed LPDC code, we save the time of reloading the check matrix. For the block length of 648,000, the times used to load data and decode data we tested with the FPGA Arria 10 are 13.0 ms and 211.2 ms. Furthermore, the average time we used to load check matrix of ATSC 3.0 LDPC codes is 11.1ms. From the Figure 5, we can see that our work keeps a high hardware processing efficiency α with the number of check matrix changing times *n* increases. Meanwhile, difference of hardware reconciliation efficiency between our proposed LDPC code and ATSC 3.0 LDPC code also increases.

**Figure 6:**
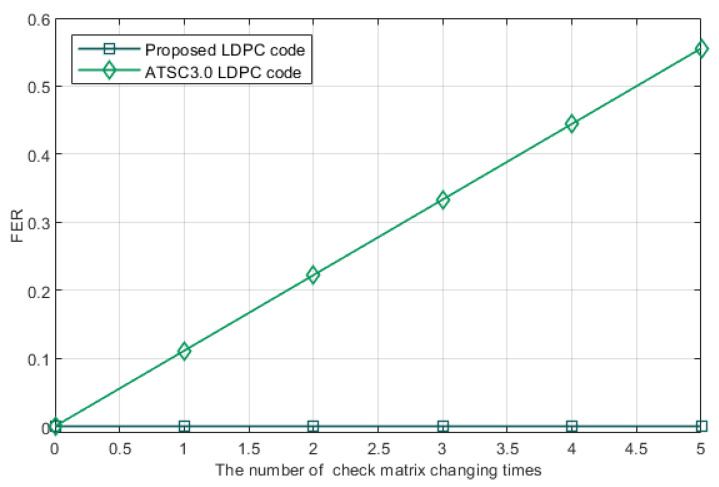
FER influenced by the number of check matrix changing times *N*. The number of data blocks that have to be processed is nine.

Frame error rate is the rate that a data block failed to be decoded. It is mainly caused by two reasons: the defect of error correcting code and decoding algorithm; the unadaptable check matrix led by the changing of SNR. The FER caused by the defect of error correcting code and decoding algorithm can be reduced to ${3.25\times 10}^{-3}$, which is far lower than the FER led by the latter reason [28]. Therefore, we only take the latter reason into account. It can be seen from Figure 6 that with the number of check matrix changes increases, our proposed LDPC code has a lower FER than the other scheme.

**Figure 7:**
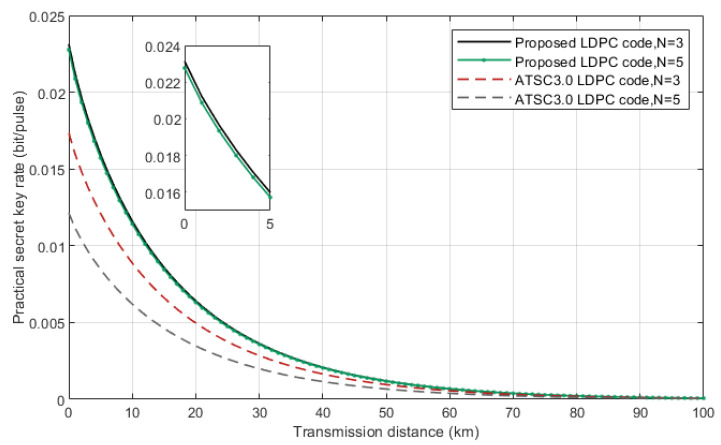
Practical secret key rate with reconciliation efficiency of 91.80% for our proposed LDPC code and 96.00% for ATSC 3.0 LDPC code. The extra parameters ε=0.01, η=0.64 and $V_{el}=0.1$.

Given the excess noise, efficiency of receiver’s detector and electronic noise at Bob’s side, we can calculate the practical secret key rate [29]. Figure 7 is the comparison of the practical secret key rate of the proposed LDPC code and ATSC 3.0 LDPC codes. As can be seen in the graph, our scheme has a better performance with same number of check matrix changes *N* and has a lower performance reduction when the *N* increases. This comes from the fact that combined action of reconciliation efficiency β, hardware processing efficiency α and FER.

## 5. Conclusions

In this study, we design a rule of proposing a RC-LDPC code with single matrix for SNRs between 0.01 and 15 to solve the problems of great variation of quantum channel noise and extremely low SNR. First, we use the discretized density evolution algorithm and differential evolution to acquire good node degree distribution pairs of LDPC codes. Then, with construction methods including PEG algorithm, random construction, quasi-cyclic extension and rate-compatible methods including extending and puncturing, we proposed a convenient and efficient construction method for designing a RC-LDPC code. Considering the number of check matrix changing times led by the change of SNR, the result shows that we have a reconciliation efficiency of 91.80%, higher hardware processing efficiency and lower FER. It has a good performance especially in an extremely unstable channel.

## Figures and Tables

**Table 1:** Related works comparison in an unstable channel. Transmission distance is 10 km and the number of check matrix changing times *N* is 3.

Reference	Hardware Resource	Secret Key Rate (bit/pulse)	Abilitiy to Cope with Channel SNR Changing
Single-matrix rate-compatible reconciliation	a, single matrix	0.0021	low
Multimatrix rate-compatible reconciliation [15]	3a, multimatrix	0.0098	low
Multimatrix corresponding to given SNRs [16]	12a, multimatrix	0.0089	low
Our proposed LDPC code	a, single matrix	0.0116	high

**Table 2:** Variable nodes degree distribution pairs for the code rate from 0.3 to 1.0.

Rate	0.3	0.4	0.5	0.6	0.7	0.8	0.9	1.0 ^1^
$\lambda_{2}$	0.0001	0.0001	0.0007	0.0001	0.0002	0.0002	0.0004	0.0005
$\lambda_{3}$	0.0047							
$\lambda_{7}$	0.5072							
$\lambda_{8}$	0.1382	0.1268	0.1044	0.0761	0.0480	0.0367	0.0281	0.0089
$\lambda_{10}$	0.3498	0.3612	0.3830	0.4119	0.4399	0.4512	0.4596	0.4787
σ	1.3868	1.1547	1.0000	0.8771	0.7809	0.7001	0.6337	0.5774
SNR	0.52	0.75	1.00	1.30	1.64	2.04	2.49	3.00
*C*	0.3072	0.4037	0.5000	0.6008	0.7003	0.8020	0.9016	1.0000

^1^ The practical rate at 1 is close to but lower than 1.

## Data Availability

Not applicable.

## Appendix A

**Table A1:** Degree distribution pairs of code rate from 0.01 to 0.1.

Rate	Degree Distribution	σ	SNR	C
0.1	$v\left(r,x\right)=0.0775r_{1}x_{1}^{2}x_{2}^{20}+0.0475r_{1}x_{1}^{3}x_{2}^{22}+0.875r_{1}x_{3}$	2.541	0.15	0.0488
	$\mu \left(x\right)=0.0025x_{1}^{11}+0.0225x_{1}^{12}+0.03x_{2}^{2}x_{3}+0.845x_{2}^{3}x_{3}$			
0.05	$v\left(r,x\right)=0.04r_{1}x_{1}^{2}x_{2}^{34}+0.03r_{1}x_{1}^{3}x_{2}^{34}+0.93r_{1}x_{3}$	5.91	0.03	0.0213
	$\mu \left(x\right)=0.01x_{1}^{8}+0.01x_{1}^{9}+0.41x_{2}^{2}x_{3}+0.52x_{2}^{3}x_{3}$			
0.02	$v\left(r,x\right)=0.0225r_{1}x_{1}^{2}x_{2}^{34}+0.0175r_{1}x_{1}^{3}x_{2}^{34}+0.96r_{1}x_{3}$	2.541	0.15	0.1008
	$\mu \left(x\right)=0.010625x_{1}^{3}+0.009375x_{1}^{7}+0.6x_{2}^{2}x_{3}+0.36x_{2}^{3}x_{3}$			
